# How Can We Treat Vulvar Carcinoma in Pregnancy? A Systematic Review of the Literature

**DOI:** 10.3390/cancers13040836

**Published:** 2021-02-17

**Authors:** Andrea Palicelli, Lucia Giaccherini, Magda Zanelli, Maria Paola Bonasoni, Maria Carolina Gelli, Alessandra Bisagni, Eleonora Zanetti, Loredana De Marco, Federica Torricelli, Gloria Manzotti, Mila Gugnoni, Giovanni D’Ippolito, Angela Immacolata Falbo, Filomena Giulia Sileo, Lorenzo Aguzzoli, Valentina Mastrofilippo, Martina Bonacini, Federica De Giorgi, Stefano Ricci, Giuditta Bernardelli, Laura Ardighieri, Maurizio Zizzo, Antonio De Leo, Giacomo Santandrea, Dario de Biase, Moira Ragazzi, Giulia Dalla Dea, Claudia Veggiani, Laura Carpenito, Francesca Sanguedolce, Aleksandra Asaturova, Renzo Boldorini, Maria Giulia Disanto, Margherita Goia, Richard Wing-Cheuk Wong, Naveena Singh, Vincenzo Dario Mandato

**Affiliations:** 1 Pathology Unit, Azienda USL-IRCCS di Reggio Emilia, 42122 Reggio Emilia, Italy; Magda.Zanelli@ausl.re.it (M.Z.); MariaPaola.Bonasoni@ausl.re.it (M.P.B.); MariaCarolina.Gelli@ausl.re.it (M.C.G.); Alessandra.Bisagni@ausl.re.it (A.B.); Eleonora.Zanetti@ausl.re.it (E.Z.); Loredana.DeMarco@ausl.re.it (L.D.M.); Federica.DeGiorgi@ausl.re.it (F.D.G.); Stefano.Ricci@ausl.re.it (S.R.); Giuditta.Bernardelli@ausl.re.it (G.B.); Giacomo.Santandrea@ausl.re.it (G.S.); Moira.Ragazzi@ausl.re.it (M.R.); 2 Radiation Oncology Unit, Azienda USL-IRCCS di Reggio Emilia, 42122 Reggio Emilia, Italy; Lucia.Giaccherini@ausl.re.it; 3 Laboratory of Translational Research, Azienda USL-IRCCS di Reggio Emilia, 42122 Reggio Emilia, Italy; Federica.Torricelli@ausl.re.it (F.T.); Gloria.Manzotti@ausl.re.it (G.M.); Mila.Gugnoni@ausl.re.it (M.G.); 4 Unit of Obstetrics and Gynaecology, Azienda USL-IRCCS di Reggio Emilia, 42122 Reggio Emilia, Italy; Giovanni.DIppolito@ausl.re.it (G.D.); AngelaImmacolata.Falbo@ausl.re.it (A.I.F.); FilomenaGiulia.Sileo@ausl.re.it (F.G.S.); VincenzoDario.Mandato@ausl.re.it (V.D.M.); 5 Unit of Surgical Gynecol Oncology, Azienda USL-IRCCS di Reggio Emilia, 42122 Reggio Emilia, Italy; Lorenzo.Aguzzoli@ausl.re.it (L.A.); Valentina.Mastrofilippo@ausl.re.it (V.M.); 6 Clinical Immunology, Allergy and Advanced Biotechnologies Unit, Azienda USL-IRCCS di Reggio Emilia, 42122 Reggio Emilia, Italy; Martina.Bonacini@ausl.re.it; 7 Pathology Unit, ASST Spedali Civili di Brescia, 25123 Brescia, Italy; laura.ardighieri@omceobs.it; 8 Surgical Oncology Unit, Azienda USL-IRCCS di Reggio Emilia, 42122 Reggio Emilia, Italy; Maurizio.Zizzo@ausl.re.it; 9 Clinical and Experimental Medicine PhD Program, University of Modena and Reggio Emilia, 41121 Modena, Italy; 10 Molecular Diagnostic Unit, Azienda USL Bologna, Department of Experimental, Diagnostic and Specialty Medicine, University of Bologna, 40138 Bologna, Italy; antonio.deleo@unibo.it; 11 Pharmacology and Biotechnology Department (FaBiT), University of Bologna, 40138 Bologna, Italy; dario.debiase@unibo.it; 12 Department of Health Science, University of Eastern Piedmont, 28100 Novara, Italy; 10034400@studenti.uniupo.it (G.D.D.); renzo.boldorini@med.uniupo.it (R.B.); 13 Pathology Unit, Maggiore Della Carità Hospital, 28100 Novara, Italy; claudia.veggiani@med.uniupo.it; 14 School of Pathology, University of Milan, 20122 Milan, Italy; laura.carpenito@unimi.it; 15 Pathology Unit, Azienda Ospedaliero-Universitaria-Ospedali Riuniti di Foggia, 71122 Foggia, Italy; fsanguedolce@ospedaliriunitifoggia.it; 16 1st Pathology Department, FSBI “National Medical Research Center for Obstetrics, Gynecology and Perinatology Named After Academician V.I. Kulakov”, Ministry of Healthcare of the Russian Federation, 117997 Moscow, Russia; A_asaturova@oparina4.ru; 17 Department of Surgical Pathology, S. Chiara Hospital, 38122 Trento, Italy; mariagiulia.disanto@apss.tn.it; 18 Unit of Pathology, Azienda Ospedaliera Universitaria Città della Salute e della Scienza di Torino, 10126 Turin, Italy; magoia@cittadellasalute.to.it; 19 Department of Clinical Pathology, Pamela Youde Nethersole Eastern Hospital, Hong Kong, China; richardwcwong@yahoo.com.hk; 20 Department of Cellular Pathology, Barts Health NHS Trust, The Royal London Hospital, Whitechapel, London E1 1BB, UK; singh.naveena@nhs.net

**Keywords:** vulva, carcinoma, cancer, HPV, lichen sclerosus, condyloma, pregnancy, cesarean, fetal, treatment

## Abstract

**Simple Summary:**

Vulvar squamous cell carcinoma (VSCC) is the most frequent malignant vulvar tumor, with a peak incidence in the 7–8th decades of life. However, VSCCs can also occur in young women. This unfortunate event is even rarer and more worrisome in pregnant women, being hard to manage for gynecologists, oncologists, and radiotherapists. Very few cases have been reported and we felt the need for an updated review on this topic. Thus, we performed a systematic literature review of VSCCs diagnosed during pregnancy, discussing the clinic-pathologic features, the implications in pregnancy outcomes, and the effects of such a diagnosis in the management of mothers and their babies.

**Abstract:**

According to our systematic literature review (PRISMA guidelines), only 37 vulvar squamous cell carcinomas (VSCCs) were diagnosed during pregnancy (age range: 17–41 years). The tumor size range was 0.3–15 cm. The treatment was performed after (14/37, 38%), before (10/37, 27%), or before-and-after delivery (11/37, 30%). We found that 21/37 (57%) cases were stage I, 2 II (5%), 11 III (30%), and 3 IVB (8%). HPV-related features (condylomas/warts; HPV infection; high-grade squamous intraepithelial lesion) were reported in 11/37 (30%) cases. We also found that 9/37 (24%) patients had inflammatory conditions (lichen sclerosus/planus, psoriasis, chronic dermatitis). The time-to-recurrence/progression (12/37, 32%) ranged from 0 to 36 (mean 9) months. Eight women died of disease (22%) 2.5–48 months after diagnosis, 2 (5%) were alive with disease, and 23 (62%) were disease-free at the end of follow-up. Pregnant patients must be followed-up. Even if they are small, newly arising vulvar lesions should be biopsied, especially in women with risk factors (HPV, dermatosis, etc.). The treatment of VSCCs diagnosed in late third trimester might be delayed until postpartum. Elective cesarean section may prevent vulvar wound dehiscence. In the few reported cases, pregnancy/fetal outcomes seemed to not be affected by invasive treatments during pregnancy. However, clinicians must be careful; larger cohorts should define the best treatment. Definite guidelines are lacking, so a multidisciplinary approach and discussion with patients are mandatory.

## 1. Introduction

Vulvar squamous cell carcinoma (VSCC) is the most frequent malignant vulvar tumor [1,2]. VSCC accounted for <1% of all female cancer cases worldwide in 2018 (estimated 44,000 new cases) [1]. As per the “Surveillance, Epidemiology, and End Results Program” (SEER) database of the United States National Cancer Institute, VSCC represented the 0.3% (*n*: 6120) of all new cancer cases and the 0.2% (*n*: 1350) of all cancer deaths in 2020 [3]. In higher-income Countries, the estimated 5-year survival rate is 50–70%, with ⁓15,000 cancer deaths/year worldwide [1].

Globally, cancer has been estimated to complicate 1:1000 pregnancies [4]. The birth-rate for women >30 years of age has been increasing while the incidence of many malignancies starts to raise during the 4th decade of life [4]. The peak incidence of VSCC is in the 7th (for Human Papillomavirus (HPV)-related VSCCs) or 8th decades of life (for HPV-independent VSCCs) [1]. However, VSCCs can also occur in young women, especially in the setting of HPV-independent VSCCs associated with lichen sclerosus or planus [1,4,5]. This unfortunate event is even rarer and more worrisome in pregnant patients, being hard to manage for gynecologists, oncologists, and radiotherapists [6]. However, very few cases have been reported. As such, we felt the need of an updated review on this topic. So, we performed a systematic literature review of VSCCs diagnosed during pregnancy, discussing the clinic-pathologic features, the implications in pregnancy outcomes, and the effects of such a diagnosis in management of mothers and their babies.

## 2. Results

### 2.1. Literature Review Results and Details of Excluded Cases

**Figure 1:**
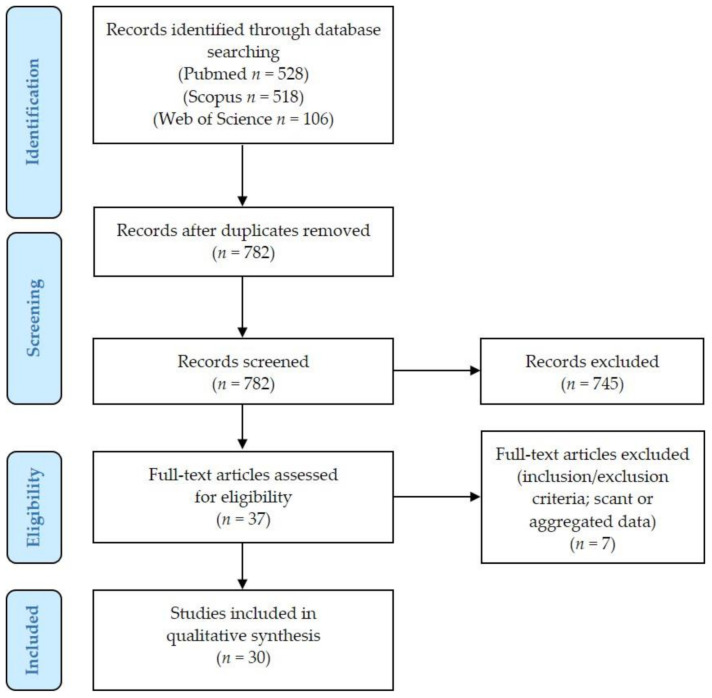
Review of the literature: Preferred Reporting Items for Systematic Reviews and Meta-Analyses (PRISMA) flow chart.

Figure 1 presents the PRISMA (Preferred Reporting Items for Systematic Reviews and Meta-Analyses) flow chart with summary of search results.

We identified 528 articles on Pubmed (https://pubmed.ncbi.nlm.nih.gov (accessed on 5 December 2020)), 518 articles on Scopus (https://www.scopus.com/home.uri (accessed on 5 December 2020)), and 106 articles on Web of Science (https://login.webofknowledge.com (accessed on 5 December 2020)) databases. After duplicates exclusion, 782 records underwent first-step screening of titles and abstracts. Of these, 37 full texts were considered for eligibility, and after reading them, 7 articles were excluded for being unfit according to the inclusion criteria or because they presented scant or aggregated data: details of some additional cases excluded from our review [7,8,9,10] are presented as Supplementary Materials. Thirty studies were finally included in the review, for a total of 37 patients diagnosed with VSCC during pregnancy [11,12,13,14,15,16,17,18,19,20,21,22,23,24,25,26,27,28,29,30,31,32,33,34,35,36,37,38,39,40,41].

### 2.2. Age and Race

The patients’ age range at presentation was 17–41 years (mean 30 years) [11,12,13,14,15,16,17,18,19,20,21,22,23,24,25,26,27,28,29,30,31,32,33,34,35,36,37,38,39,40,41] (Table 1). The majority of cases was diagnosed in the United States (15 cases) [18,23,27,29,30,31,35,36,37,40,41], 12 in Europe (5 United Kingdom, 3 France, 2 Germany, 1 Belgium, 1 Holland) [11,12,13,17,20,22,24,26,33,38,39], 6 in Asia (1 Iran, 1 Nepal, 1 Turkey, 1 Malaysia, 1 Saudi Arabia) [14,16,19,21,32,34], 2 in Australia [28] and 2 in Africa (1 Congo, 1 Nigeria) [15,25].

Eight patients were black [15,25,27,29,31,36,41], 6 were white/Caucasian [13,17,22,30,31,40], and 6 were of different Asian races [14,16,21,26,32,34]. The race of the remaining 17 patients was unclear.

### 2.3. Primary Tumor (T)

Globally, the anterior/upper part of the vulva was involved in 22/37 (60%) cases [13,14,19,21,22,23,24,25,26,27,28,29,30,31,32,34,36,38,39], and the posterior/lower portion in 10/37 (27%) cases [18,20,25,28,31,35,36,41] (Table 1, Table S1). Nine lesions were centered in the median anterior/upper (6 cases) [21,26,30,32,38] or median posterior/lower (3 cases) [18,35] part of the vulva, extending bilaterally. One case seemed confined to the mid vulvar part [36]. The clitoris/periclitoral area was infiltrated in 14/37 (38%) cases [13,19,21,23,24,26,28,30,31,32,34,38,39], labium majus in 18/37 (49%) cases [13,15,19,20,21,22,23,29,31,32,33,34,36,37,40,41], labium minus in 11/37 (30%) cases [13,14,17,20,21,24,27,28,36], the posterior fourchette in 3 (8%) cases [18,20,28]. Unilateral [14,15,17,19,20,22,23,24,27,29,31,33,34,36,40,41] and bilateral cases [13,18,21,23,24,25,26,28,30,31,32,35,36,37,38,39] were almost equally distributed (17/37, 46% and 18/37, 49% respectively) (unavailable information for the remaining cases). Eight unilateral tumors occurred on the right hemivulva [15,17,19,20,22,31,36,40], and 9 on the left hemivulva [14,23,24,27,29,33,34,36,41].

Furthermore, 6/37 (16%) VSCCs were multifocal, either unilateral (1 case, right) [20] or bilateral (5 cases) [23,24,28,31,36]. Each multifocal case developed 2 foci of VSCCs, either synchronous (4/6 cases, 67%) [20,28,31,36] or metachronous (2/6 cases, 33%). In 2 (5%) cases the whole vulva was involved by single [25] or multiple lesion(s) [28]. In 3 (8%) cases, location was unreported [11,12,16]. As regards the concomitant evidence of vulvar condylomas (VCs) see Section 2.16.

The size of vulvar lesions ranged from 0.3 to 15 cm (mean 3.6 cm) [11,12,13,14,15,16,17,18,19,20,21,22,23,24,25,26,27,28,29,30,31,32,33,34,35,36,37,38,39,40,41]. VSCCs were described as raised (5 cases) [18,22,24,31,41], verrucous/exophytic/papillary/proliferative/fungating (11 cases) [17,20,21,25,28,32,34,35,36,40], nodular (2 cases) [15,24], solid (1 case) [20], firm plaque/flat (2 case) [22,36], ulcerated (18 cases) [13,14,17,20,21,22,26,27,28,29,30,33,35,36,38,39,40], bleeding (1 case) [15], necrotic (3 cases) [20,21,26], painful (4 cases) [17,23,24,27], pigmented (1 case) [18], or abscess-like (1 case) [31].

Vaginal invasion (lower third) occurred at presentation in 3 (8%) cases [20,35,41]. The anus was infiltrated in 3 (8%) cases (2 at presentation, 1 with disease progression) [20,25,35]: 1/3 (3%) tumors also invaded the lower rectum [20]. No primary VSCCs invaded cervix or bladder at presentation, despite a patient developed acute urinary retention with disease progression [25] while another woman revealed a tumor extending within 1 mm of the urethra (1 case, 3%) [28]. Palpable inguinal lymph nodes (LNs) were found in 12/37 (33%) cases at presentation, either bilateral (6 cases) [13,20,21,27,32,35] or unilateral (5 cases: 3 left [31,38,39], 2 right [15,28]); in 1 case it was unclear if the palpable LNs were bilateral or unilateral [31].

### 2.4. Clinical Presentation

According to the time of discovery of the vulvar lesions, the patients can be grouped into:

1. **Incidental discovery at the time of delivery (5/37 cases, 13%)**: labor occurred at 29 gestational weeks (GW) in 1 case [22], and at 7th post-term day in another woman [20] (unclear timing in 3 cases) [27,35,36].
2. **Pre-conceptional evidence of a vulvar lesion (4/37 cases, 11%)**. The vulvar lesion was evident 1 [15], some [17], 12 [28], and 15 months [25] before conception, respectively. However, the diagnosis of VSCC was made during the 1st trimester (1 case: 2nd month) [28] or even during the 2nd trimester of pregnancy (3 cases: 19 GW [17]; 16 GW [25]; 5th month [15]). A tumor was diagnosed as a condyloma 6 months before conception, while subsequent biopsy at 29 GW revealed a VSCC [25]: this discrepancy may be due to misdiagnosis of the first biopsy or malignant transformation during pregnancy. Another VSCC was diagnosed during the 5th month of pregnancy in the site where a small itchy lesion of unknown diagnosis had been excised 1 month before conception [15]. In the remaining 2 cases, it was unclear if the lesion was underestimated by clinicians or not presented to the gynecologists’ attention [17,28].
3. **Discovery during pregnancy without pre-conceptional evidence of vulvar lesions (25/37 cases, 68%)**. The VSCCs were identified during the 1st (2/25 cases, 8%) [14,35], 2nd (14/25 cases, 56%) [13,16,18,23,24,28,29,31,32,33,36,37,38,39], or 3rd trimester of pregnancy (9/25 cases, 36%) [11,12,19,21,26,30,31,36,41].
4. **Peri-conceptional evidence of a vulvar lesion (3/37 cases, 8%).** Vulvar lesions were diagnosed as VSCCs at 10 GW [24], 24 GW [34], and 6.5 months after conception [40], respectively. However, patients reported evidence of vulvar lesions within a month before or after the probable day of conception.

In most cases, it was unclear if the vulvar lesion was first identified by patients or clinicians. Additional symptoms or pregnancy complications (see Section 2.18.) may have led the patients to seek clinical evaluation.

**Vulvar symptoms**: Vulvar itching was suffered by 5/37 (13%) patients at presentation: it started during pregnancy in 3/5 (60%) cases (22 GW [21], 28 GW [26], 19 GW [29]). 2/5 (40%) patients had 10-year- [24] and 1-year-history of vulvar pruritus [34]. Additional symptoms included: vulvar swelling (9- and 10-weeks history) (2 cases) [21,31], vulvar soreness/pain (3 cases) [27,31,34], vulvar bleeding (1 case) [29], and suppurative vulvar cellulitis (1 case) [30].

**Vaginal symptoms**: Three out of 37 (8%) women had vaginal discharge [25,26,41] which was bloody in 2/3 cases [26,41]. One woman reported experiencing vaginal itching and burning [31].

### 2.5. Risk Factors

For HPV status and vulvar inflammatory conditions see Section 2.16.

A smoking history was evident in 3/37 (8%) cases [18,20,22] (1 heavy-smoker [20]; 1 moderate-smoker for 2 years [22]; 1 smoker not otherwise specified, NOS), being excluded in 3 additional cases [17,28].

Three patients (8%) had a history of sexually transmitted disease [29,36]: syphilis and lymphogranuloma venereum (positive Frei test; Donovan bodies on vulvar scraping) (1 case) [36]; syphilis and granuloma inguinale (1 case) [36]; *Chlamydia* infection during pregnancy (1 case) [29]. In 12 women, sexually-transmitted diseases were excluded (when specified, variably including syphilis, Human immunodeficiency virus (HIV), *Herpes simplex* virus, or *Hemophilus ducreyi*) [14,18,20,21,23,25,26,27,28,40,41].

Drug abuse was admitted by 2/37 (5%) patients: cocaine in the past [27]; cocaine and heroin during pregnancy [29]. Four women denied drugs abuse [18,20,28] and data were unavailable for the remaining cases. A patient had a history of pregnancy-related recurrent bone marrow hypoplasia with pancytopenia: immunofluorescent studies for Behçet’s disease resulted negative [26]. Further reported clinical features included: treatment for pellagra 2 years before presentation (1 case) [40]; obesity and severe varicosities (1 case) [35]; insulin-dependent diabetes (1 case) [24].

### 2.6. Pap Smear

A woman (1/5, 20%) who underwent a Pap smear at presentation received a diagnosis of “Atypical squamous cells of undetermined significance” (ASCUS)/“Atypical glandular cells” (AGC) [27]. Her previous Pap tests were negative, but she had condylomas affecting the entire vulva at presentation, and biopsies also revealed high-grade squamous intraepithelial lesions (H-SIL) of cervix and anus. The remaining 4/5 cases resulted negative [21,28,31,35]. Another patient had a history of VCs and cervical dysplasia treated with cryosurgery at age 22 years (7 years before presentation) [29]: cervical cytology samples were not taken at presentation, as for 3 women which never had abnormal Pap smears in their history [18,20,24].

### 2.7. Biopsy

In 32/37 cases (87%), the vulvar lesion was biopsied during pregnancy (25/32 cases, 78%) (range 8–36 GW; mean 23 GW) [11,12,13,17,18,19,21,23,24,25,26,28,29,30,31,33,34,35,36,38,39,40,41], during delivery (when it was discovered) (5/32 cases, 16%) [20,22,27,35,36], or after delivery (2 weeks postpartum: 1 case, unreported time: 1 case) [14,31] (Table S2). In 2 cases, 2 biopsies were performed (33 GW and 4 weeks after delivery [26]; 10 GW and 22 GW [24]).

### 2.8. Fine Needle Aspiration Cytology of Lymph Nodes

Fine needle aspiration cytology (FNAC) of suspicious inguinal LNs was performed in 2/37 (5%) cases (15 GW; after delivery) [13,31], resulting as positive for malignant cells in both cases and addressing to bilateral inguinal-femoral lymphadenectomy in 1 case (15 metastatic LNs were identified) [31].

### 2.9. Timing of Treatment

In 14/37 (38%) cases, treatment was entirely carried on 0–84 days after delivery (mean 27 days), performing 1 (11 cases) [11,14,15,19,20,22,26,27,31,32,35], 2 (2 cases) [31,36] or 3 [26] different procedure(s) (Table S2). It was decided to perform surgery after delivery for the following reasons:

1. **Discovery during delivery** (5/14 cases, 35%) [20,22,27,35,36]
2. **Spontaneous vaginal delivery probably delaying surgery** (1/14 cases, 7%): labor occurred 1 month after presentation [36]
3. **Cesarean section (CS) was rapidly performed before treating VSCC** (4/14 cases, 29%), as the woman was in the 3rd trimester (32–37 GW) [11,15,19,31].
4. **Misdiagnosis/underestimation by clinicians/patients** (4/14 cases, 29%). Clinicians prescribed typical ointments and antibiotics for a vulvar lesion noted during the 3rd month of pregnancy: the correct diagnosis was made after delivery [14]. A lesion that was evident during the 2nd trimester of pregnancy was not biopsied until 3 months after spontaneous vaginal delivery [31]. In 1 case, it took to the clinicians 5 weeks (33–38 GW) to understand the tumor nature of the vulvar lesion and perform a vulvar biopsy: the diagnosis was low-grade squamous intraepithelial lesion (L-SIL; vulvar intraepithelial neoplasia, VIN1) + H-SIL (VIN2). An elective CS was performed at 38 GW; unfortunately, the patient and her family were non-compliant: the lesion grew and extended in the meanwhile [26]. A woman presented at 16 GW with a vulvar lesion and she was referred to the nearby district hospital for further investigations; however, she defaulted and presented instead during labor at term [32].

In 10/37 (27%) cases, the treatment was entirely carried on during pregnancy as 1 (6 cases) [17,28,34,35,39] or 2 procedure(s) (4 cases) [24,29,33,38]. When reported, the treatment was started 0–18 weeks after presentation, during the 2nd (7 cases) or 3rd trimester of pregnancy (1 case) (15–28 GW; mean 22.5): delivery occurred 8–18 weeks after the last surgical procedure (mean 12.5 weeks) [24,28,29,33,34,38,39].

In 11/37 (30%) cases, the treatment was split into different procedures which were carried on before and after delivery: the diagnosis was made during the 2nd (6 cases) [13,18,23,28,36,37] or 3rd trimester [12,21,30,40,41], while treatment was started during pregnancy 0–5 weeks after diagnosis (mean 2.1). Delivery occurred 3–18 weeks (mean 9 weeks) after treatment, at 29–38 GW (mean 34 weeks). Postpartum treatment started at 2–28 weeks (mean 5.7) after delivery. The treatment of one patient was unclear [16], while one was not treated [25].

### 2.10. Surgery

Table S2 details the type and timing of surgery, including:

1. Local excision/excisional biopsy (7/37 cases, 19%): 5 during pregnancy (18–33 GW) [17,21,29,33,38]; 2 after delivery (3 and 9 weeks postpartum) [22,23]. Complications: superficial wound breakdown 4 weeks later [21].
2. Partial vulvectomy (4/37 cases, 11%): during pregnancy in all cases (15 GW to 4 months before delivery) [18,24,37]. In 1 case, 2 partial vulvectomies were performed (15 and 22 GW) [24]. Complications: lymphocele (10 days after surgery) (1 case) [24]; right inguinal abscess, right lymphocele, and perineal mycosis after the first partial vulvectomy [24].
3. Hemivulvectomy (3/37 cases, 8%): 1 during pregnancy (26 GW) [24]; 2 after delivery (NOS) [11,15].
4. Vulvectomy (3/37 cases, 8%): 2 during pregnancy (22 GW [38]; 6.5 gestational months [40]); 1 after delivery (6 months) [41]).
5. Surgery NOS (2/37 cases, 5%) [12,16] (34 GW+ re-excision after delivery) (unclear when performed).
6. Radical vulvectomy (22/37 cases, 60%):
  (a) Ten cases were performed during pregnancy (17–29 GW, mean 28) [23,28,29,30,33,34,35,36,39]. Complications: persistent watery vaginal discharge (intra-amniotic instillation of methylene blue ruled out rupture of membranes) (1 case) [30]; bilateral groin seromas after 12 days (resolved after aspiration and pressure dressing) (1 case) [28]; pain and swelling of left leg for a right inguinal wound seroma after 18 days (treated with drainage and bed rest), and cellulitis of left leg at 37 GW (treated with antibiotics) (1 case) [29]; initial vulvar edema [23].
  (b) Twelve cases were performed after delivery [18,19,21,26,27,31,32,35,36,37] (1–12 weeks postpartum, mean 7 weeks). Complications: fever (39.4 °C) on 2nd postoperative day [26]; hematoma in the right groin wound (drainage on 2nd postoperative day) [27]; necrosis of distal flaps, requiring surgical debridement [31].

### 2.11. Sentinel Lymph Node

Sentinel lymph node (SLN) resection was performed in 4/37 (11%) cases (3 bilateral, 1 right) [11,12,17,18]. In 2 cases, the procedure was made during pregnancy [12,17], in 2 others postpartum [11,18]. In 1 case, ^99m^Tc (dosages of 10.73 and 10.15 and 11.07 and 9.9 MBq on 4 sites of injection) was used to identify SLN by scintigraphy, avoiding patent blue during pregnancy [17]. In another case, isosulfan blue was used postpartum to identify bilateral SLNs declining ^99m^Tc for patient’s concern about possible radioactive exposure while breast-feeding [18]. SLN resulted metastatic in 1/4 (25%) cases: it was unclear if subsequent right inguinal-femoral lymphadenectomy found further metastatic LNs [11]. Additional right groin LN-sampling was made in a negative-SLN case: 9 reactive LNs were resected [18].

### 2.12. Lymph Node Sampling/Lymphadenectomy

LN-sampling was performed in 3/37 (8%) cases, including right groin LNs (postpartum) [18], internal/external iliac LNs (during CS at 38 GW) [31], and left superficial LN (22 GW) [38]. LNs resulted negative in all cases.

Lymphadenectomy was performed in 28/37 cases (76%), either bilateral (22/28 cases, 79%) [14,19,21,23,26,27,28,29,30,31,32,33,34,35,36,39,40], or unilateral (4/28 cases, 14%) (3 right [11,15,24], 1 left [24]). It was unclear if lymphadenectomy was uni- or bi-lateral in 2 cases (7%) [37,41]. When reported, the number of resected LNs ranged from 5 to 61 (mean 21). The following LN-sites were removed: inguinal-femoral (18 cases) [11,14,19,21,23,24,26,27,28,29,31,33,34,39,40,41]; inguinal-femoral, external iliac and obturator (1 case) [35]; inguinal and pelvic (1 case) [32]; superficial and deep inguinal-femoral, Cloquest’s, external iliac, obturator, hypogastric, common iliac, aortic and caval (3 cases) [36]; femoral and iliac (1 case) [35]; inguinal superficial (1 case) [30]. Further data on metastatic LNs are provided in Section 2.15.

### 2.13. Chemotherapy

As one patient did not undergo surgery for advanced stage disease at presentation, chemotherapy (carboplatin-vinorelbine) was started at 18 GW: as to local disease progression, chemoradiation was administered 9 days after delivery (cetuximab + cisplatin; then only cetuximab for radiotherapy-induced pancytopenia). Five months after diagnosis, the patient died due to pulmonary metastases [13]. Another patient underwent adjuvant chemoradiation (3 courses of vincristin 1 mg/m^2^ and cisplatin 50 mg/m^2^) for a stage 3 VSCC at least 21 days postpartum: no evidence of disease was found 7 months after diagnosis [21]. No chemotherapy was administered to the remaining 35 patients.

### 2.14. Radiotherapy

Radiotherapy was administered to 10/37 (8%) patients [13,19,20,21,26,28,31,35,40,41], being performed after delivery in 9/10 (90%) cases. Neoadjuvant brachytherapy (described as radium bombs into the vulvar and inguinal areas) was administered to a patient around the 7th month of pregnancy, obtaining partial response; after delivery, neoadjuvant roentgen therapy (7 cycles, 1764 r. units) was administered 17 days postpartum, seeming to achieve complete response: no evidence of disease was found 5 years after subsequent vulvectomy + inguinal lymphadenectomy [41].

Radiotherapy was performed in 2 women who did not undergo surgery for locally advanced VSCC [13,20]. In the first case [13], chemoradiation was administered after delivery (and few courses of prenatal neoadjuvant chemotherapy): radiation treatment details were not provided. The woman died 5 months after diagnosis for metastatic disease. In the second case [20], after CS, the patient underwent palliative radiotherapy (50 Gy in 25 fractions) achieving only partial response: the woman died of disease progression 11 months after diagnosis [20].

In 7 cases, adjuvant radiotherapy was administered from >1 week to >21 weeks postpartum [19,21,26,28,31,35,40]: scant details were provided. Complications included: superficial wound breakdown [31]; left-sided lymphocyst (positive for malignant cells on FNAC) [26]; subcutaneous abscess in inguinal wound area, low-grade thrombophlebitis, and cellulitis of the right leg and foot [40].

### 2.15. Pathological Examination and Stage

No particular morphological features of VSCCs were reported on histopathological examination. Data (including photographic documentation) were frequently scant or lacking, especially in old reports. Twenty-five VSCCs were graded (Table 2): 11 G1 (44%) (in 1 case G3 spindle cells were found in the nodal metastasis) [17,18,19,21,22,24,32,34,35,38], 9 G2 (36%) [11,23,27,28,29,30,31,33], 5 G3 (20%) [13,15,20,26,35]. Perineural invasion was excluded in 1 case [17] (unclear data for the other cases).

Four radical vulvectomy specimens (4/37, 11%) revealed only H-SIL (1 VIN2, 1 CIS) (2 cases) [18,29] or no residual tumor (2 cases) [33,41]. In 3 cases (1 local excision/excisional biopsy [21], 1 surgery NOS [12] and 1 partial vulvectomy [18]), the surgical margins were involved, addressing further treatment. In 2/3 cases, the radical vulvectomy margins were involved by H-SIL [28,31]: adjuvant radiotherapy was administered to a patient [28]. No recurrence of VSCC was found in these 2 cases (28 months and 16 months after primary surgery, respectively).

According to the 8th edition of the American Joint Committee on Cancer (AJCC) classification [2], 21/37 (57%) cases were stage I, including 4 IA (11%) [22,24,36,38], 15 IB (41%) [12,14,17,18,23,24,25,28,29,30,34,36,39,40], and 2 stage I NOS carcinomas (5%) [33,37] (Table 2). Two VSCCs were stage II (5%) [35,41], 11 stage III (30%) [11,15,16,19,21,26,27,28,31,35], and 3 stage IVB (8%) [13,20,32].

The pathological analysis of LNs was performed in 33/37 (89%) cases [11,12,13,14,15,17,18,19,21,23,24,26,27,28,29,30,31,32,33,34,35,36,37,38,39,40,41]. Globally, the number of resected LNs ranged from 1 to 61 (mean 13) [12,13,17,18,23,24,27,28,36,38]. 22/37 (67%) cases resulted pN0 [12,14,17,18,23,24,28,29,30,31,33,34,35,36,37,38,39,40,41], 11/37 (33%) cases pN+ [11,13,15,19,21,26,27,28,31,32,35]. The number of metastatic LNs ranged from 1 to 17 [1,2,3,4,5,6,7,8,9,10,11,12,13,14,15,16,17,18,19,20,21,22,23,24,25,26,27,28,29,30,31,32,33,34,35,36,37,38,39,40,41]. Metastatic LNs were inguinal/femoral (10 cases) [11,13,15,19,21,26,27,28,31,35]; inguinal, external iliac, obturator (1 case) [32]. When reported, positive LNs were bilateral in 5 cases [26,27,31,32,35], unilateral in 3 cases [11,15,19]. In 1 pN+ case, suspicious lomboaortic LNs were additionally detected by imaging [13]. Finally, suspicious inguinal and pelvic LNs extending to both internal iliac groups were found on magnetic resonance imaging in 1 case (cN+) [20]. The size of LN-metastases and the evidence of extranodal extension were usually not reported. Lymphovascular invasion was described in 2 high-stage tumors at [26,32], and excluded in 6 stage IA/B VSCCs (pN0/pNx) [14,17,22,23,30,38].

### 2.16. HPV Status and Cancer Precursors

HPV testing with hybrid capture technique (Digene, Gaithersburg, MD, USA) was tested only in 1 VSCC, resulting positive for high-risk HPV-subtypes (16, 18, 31, 33, 35, 45, 51, 52, 56) [27]. Conversely, a VSCC was recently reported as HPV-independent [11]. It is difficult to clearly determine if the remaining cases were HPV-associated or not (VSCC, NOS) [1]. Immunohistochemical examination (also including p53 and p16 immunomarkers) or molecular analysis were not performed in any of the reported VSCCs or SIL/VIN. In none of the cases treatment was guided by the HPV status of the tumor.

Despite an evaluation of the HPV status is lacking in most VSCCs, HPV-related lesions (condylomas/warts; HPV infection; genital H-SIL) were globally reported in 11/37 (30%) cases at presentation or in previous history (Table 2) [18,21,23,24,25,27,28,29,31,36,40]. These lesions may represent an indirect sign of a HPV-associated VSCC, despite they are not specific (especially if the HPV infection was present only in patient’s history). Moreover, morphologic evaluation shows limitations in predicting the HPV status of precursor lesions and it cannot be always reliable; however, immunohistochemistry was not performed in any cases (see Discussion). Finally, H-SIL was also associated with inflammatory conditions in 2 cases [18,23].

Eight out of 37 (22%) women were affected by VCs [21,24,25,27,28,29,36,40]. VCs were just reported in patient’s history (2 cases) [21,29] or diagnosed at presentation in patients without a history (4 cases) [25,27,36,40]; finally, 2 women had a history of VCs but presented with new lesions [24,28]. Subsequent malignant transformation may have occurred in a newly diagnosed VC [25]. At presentation, VCs were multiple and bilateral [24,40], or affected the entire vulva (or most of it) [27,28]: a new VC arose during pregnancy after the diagnosis of other carcinomatous foci [24].

Vulvar H-SIL was found in 7/37 (19%) cases (1 moderate, 3 severe, 1 NOS, 1 bowenoid, 1 in situ carcinoma) [18,23,24,27,28,31,36]: 4/7 (57%) patients also showed VCs at presentation [24,27,28,36].

Nine patients (9/37, 24%) had a background of inflammatory conditions (Table 2) [13,14,17,18,22,23,24,30,39].

Lichen sclerosus affected 6/37 (16%) patients (4–17 years before presentation) [17,18,23,24,30,39]. Three cases were biopsy-proven [23,24,30]. Treatment had included clobetasol (2 cases) [18,23], corticosteroids NOS (1 case) [24], or estrogens (topic and per os) + radiotherapy (9 doses; 300 rads/dose) [39]. In 2 cases, lichen sclerosus/kraurosis vulvae was particularly severe [17,39], causing labial fusion caudal of the clitoris in a woman [17]. No signs of HPV-infection were found in a patient with lichen sclerosus [17] while H-SIL was identified in the vulvectomy specimen of 2 patients with lichen sclerosus [18,23].

No HPV-independent VIN (differentiated VIN) was clearly described, despite a vulvar leukoplakia due to hypertrophic lichen planus and hyperplastic vulvar dystrophy without atypia or signs of HPV-infection was reported [22]. One patient was treated with topical corticosteroids from 5 to 12 GW (3 weeks before presentation) for recurrent vulvar psoriasis [13]. Finally, a patient complained of progressive vulvar itching for 17 years: vulvar hyperplasia and chronic dermatitis were found on a biopsy performed 2 years before presentation [14].

Three cases of leukoplakia NOS were described [29,34,38].

In 17/37 (46%) cases, a possible vulvar precursor lesion was not identified neither at presentation nor in patient’s history [11,12,15,16,19,20,26,28,31,32,33,35,36,37,41]: 2/17 women showed vaginal itching ± burning during pregnancy, but it was unclear if it was related to an inflammatory condition [26,31].

In 3/37 (8%) VSCC-patients, HPV-related precursor lesions were found in the anus, including Bowenoid disease of perianal skin (associated with a 6-year history of VCs and leukoplakia) [29], perianal condylomas (evident at presentation) [36] and H-SIL (biopsy-proven, associated with VCs) [27]. Vaginal warts were identified only in 1 case (3%) [28], while cervical H-SIL was diagnosed in 2 cases (5%) [27,28]. Finally, a patient developed a stage 2 squamous cell carcinoma of cervix 9 years after treatment for VSCC: the cervical primary was irradiated; 16 years after surgery for VSSC, small areas of vaginal in situ carcinoma were widely excised [36].

### 2.17. Follow-Up Data

Follow-up data were available for 33/37 (89%) cases (Table 3) [11,12,13,14,15,17,18,19,20,21,22,23,24,25,26,28,31,32,33,34,35,36,37,38,39,40,41]: the range of patients’ follow-up was 2.5–204 (mean 33) months. Globally, 14/37 (38%) cases recurred or showed disease progression, including 13 invasive VSCCs [11,13,15,18,19,20,23,24,25,26,31,32,36] and a SIL/VIN recurrence [28]. 

Two out of 14 (14%) cases were classified as new metachronous vulvar primaries, both diagnosed during pregnancy: the interval of time from excision of the first lesion to the subsequent tumor was 7 [24] and 11 weeks [23], respectively.

The time to recurrence/disease progression of the remaining 12 cases (12/37, 32%) ranged from 0 to 36 (mean 9) months after treatment: 4 cases recurred locally in the vulva [11,18,28,36], 1 locally and in the lungs [13], 1 in the abdomen, anus, and inguinal region [25], 4 in the inguinal/groin region [15,19,20,26], 1 in the lumbar vertebrae and periaortic lymph nodes [31], and 1 in an unknown site [32].

Eight women died of disease (22%) [13,15,19,20,25,26,31,32]: exitus occurred 2.5–48 (mean 13.9) months after diagnosis. The final cause of death was reported in 3 cases: sepsis (2 cases) [25,32]; cardiorespiratory decompensation for pulmonary metastases (1 case) [13]. In all these patients, the disease progressed or recurred 1.5 to 36 (mean 12) months after primary treatment [13,15,19,20,25,26,31,32]. Two stage III VSCCs recurred in inguinal LNs [19] or lumbar vertebrae/periaortic LNs [31] (36 and 16 months after surgery, respectively). 6/8 (75%) patients underwent disease progression [13,15,20,25,26,32]. A patient with stage IB disease declined treatment, so it progressed to IVB disease (progressive swelling of the right leg, sepsis along the CS-scar, multiple intra-abdominal abdominal, inguinal and anal involvement) 7 weeks post-CS [25]. Three stage IVB VSCCs recurred in vulva and lungs 13 weeks and 5 months after diagnosis [13], in groin LNs 3 months after radiotherapy [20] and in unreported sites in the last case [32]. The 2 remaining cases (at least stage IIIA) progressed in the inguinal region (after radiotherapy treatment in 1 case) [15,26].

Two patients (5%) were alive with local disease recurrence ≥6 months after surgery: the 1st case recurred once, the 2nd twice [11,18].

Twenty-three patients (62%) showed no evidence of disease at the end of follow-up (range 6–204 months, mean 42.6 months) [12,14,17,21,22,23,24,28,31,33,34,35,36,37,38,39,40,41] including the 2 women with metachronous VSCCs [23,24], a patient with recurrent VSCC, stage 2 cervical squamous cell carcinoma and vaginal in situ carcinoma [36], and 1 relapsing vulvar H-SIL [28].

### 2.18. Pregnancy Course

Seven women (19%) were nulligravid [18,22,31,33,38,39,41], while 26 patients (70%) had multiple previous (2–9) pregnancies (Table 4) [11,13,14,15,19,20,21,23,24,25,27,28,29,30,31,32,34,35,36,37,40]. Delivery of the ongoing pregnancy occurred from 29 GW to 1 week after term.

Delivery was vaginal in 14/37 cases (38%) [16,22,23,24,29,30,31,32,34,35,36], occurring spontaneously at least in 6 cases [30,31,32,35,36]. In 1 case, labor was induced for disease recurrence [23].

Unilateral or bilateral episiotomies were additionally performed in 6/14 cases (43%) [24,29,30,34,35,36]. Low forceps under spinal anesthesia was used in 1 case, and posterior colporrhaphy was also performed [36].

CS was performed in 22/37 (59%) cases [11,13,14,15,17,18,19,20,21,24,25,26,27,28,31,33,37,38,39,40,41]. Further CS-details included: emergency CS (1 case for sepsis) [13], elective (3 cases) [18,25,26], low segment CS (2 cases) [20,39], CS + surgery [31,37]. In 1 case, the delivery type was unknown [12]. Pregnancy was single in 25/37 (68%) cases [13,14,18,20,21,22,23,24,25,26,28,29,30,31,32,33,34,35,36,38,39,40,41], multiple (twin) in 2/37 (5%) cases (dichorial, diamniotic in 1) [15,17] (unclear in remaining cases).

All the babies were delivered alive, except for one stillborn [36]; a newborn was resuscitated and transferred to a special care unit after vaginal delivery [22]. Six newborns were females [13,20,31,33,36,41], 9 males [18,22,24,25,26,28,38,39,40] (unclear sex in 22 cases) [11,12,14,15,16,17,19,21,23,24,27,28,29,30,31,32,34,35,36,37].

Baby weight ranged from 1000 to 3990 g (mean 2697 g) [13,18,20,21,22,24,25,29,30,31,32,34,36,38,39,40]: 7 babies weighted <2500 g [13,18,22,24,25,36,38].

In addition to vulvar scars and other complications of treatment, relevant events during pregnancy included: *Chlamydia* infection, cocaine and heroin abuse (1 case) [29]; suppurative vulvar cellulitis at 25–27 GW (1 case) [30]; subsequent foci of VSCC (2 cases) (34 GW [23]; 22 GW [24]) ± VC (27 GW) [24]; sepsis (*Pseudomonas aeruginosa*), uncontrolled by antibiotic therapy (1 case) [13]; obstetrical complications/prolonged pregnancy (4 cases) [17,20]; hemorrhage (2 cases) [26,38]; evidence of uterine fibroids [26]; hypertension and proteinuria at 34 GW [38]; abnormalities of fetal heartbeat and membrane rupture [24]. A patient developed postpartum pyelitis, and abscess of left axillary space for 15 days [41].

Five (14%) patients had subsequent pregnancies after treatment of VSCC [35,36,38,39]: babies were healthy and delivered vaginally (3 cases) [35,36] or by CS (2 cases) [38,39] from 20 to 46 (mean 31) months after primary treatment of VSCC. 1/5 (20%) women aborted 18 months after VSCC [36].

## 3. Discussion

Pregnancy is characterized by excess of circulating blood volume, secretion of sex-/growth- hormones, and immunosuppressive status: all these physiologic changes could favor cancer growth or progression [1,2,3,4,5,6,7,8,9,10,11,12,13,14,15,16,17,18,19,20,21,22,23,24,25,26,27,28,29,30,31,32,33,34,35,36,37,38,39,40,41,42]. To our review, their potential effect on VSCCs was not investigated by experimental studies as the reported cases were all clinic-pathologic case reports/small series. The effects of cancer on pregnancy may include mechanical uterine compression, tumor blocking of delivery route, cancer-related inflammatory cytokine production, or metastases to fetus or placenta [42]: none of them was reported or investigated by the articles included in our series.

As per the 2021 guidelines of the United States National Comprehensive Cancer Network (NCCN-g) [43], the best treatment of early-stage VSCC is represented by radical local excision (with 1-cm grossly free margins) and unilateral or bilateral inguinofemoral lymphadenectomy (IF-LND) (or SLN biopsy in selected patients). Simple partial vulvectomy should be performed for pT1a tumors with ≤1 mm of stromal invasion: in these cases, IF-LND or SLN biopsy can be omitted if LNs are clinically negative, as the risk of lymphatic spread is <1% [43]. Conversely, radical partial vulvectomy with IF-LND (preceded or not by SLN biopsy) is reserved for more invasive (stage IB–II) VSCCs, as to the >8% risk of lymphatic involvement [43]. Groin treatment should be performed for tumors > pT1a also according to the European Society of Gynecological Oncology (ESGO-g) [44].

For the NCCN-g, women with unifocal VSCC of <4 cm, without suspicious groin nodes on clinical/imaging examination, are “candidates” for SLN biopsy [43]; this procedure is also “recommended” by the ESGO-g [44]. Conversely, larger or multifocal VSCCs require IF-LND by separate incisions [43,44]. SLN procedure should be performed prior to the VSCC excision to avoid disrupting the lymphatic network between the primary tumor and the SLN: if SLN is not found (method failure), IF-LND should be performed [43,44].

Minor differences may be found between NCCN-g and ESGO-g. To the ESGO-g, ipsilateral IF-LND should be performed when a metastasis of any size is found in the SLN [44]. Conversely, to the NCCN-g, IF-LND can be omitted if a single positive SLN reveals a ≤2 mm metastasis and in this case the patients undergo external beam radiation therapy ± chemotherapy [43].

Despite a < 3% risk of contralateral metastases, a unilateral IF-LND or SLN biopsy is appropriate for unifocal lateral tumors <4 cm, located ≥2 cm (NCCN-g) or >1 cm (ESGO-g) from the vulvar midline and in the setting of clinically reactive inguinofemoral LNs: if metastatic LNs are found, resection or radiation therapy of the contralateral groin is recommended by the NCCN-g, while contralateral IF-LND may be performed according to the ESGO-g [43,44]. If the tumor is closer to the vulvar midline, the abovementioned procedures should be bilateral [43,44].

The optimal management of the groin for bulky, proven metastatic LNs is unclear (IF-LND vs. the removal of isolated positive LN) especially for unresectable or pT3 VSCCs (NCCN-g/ESGO-g): lymphadenectomy for stage III-IV disease is individualized (NCCN-g) [43,44].

Unfortunately, despite these indications, there are no clearly reported guidelines concerning the treatment of VSCCs diagnosed during pregnancy [5,6]. As such, a multidisciplinary approach is mandatory: mother, fetus and malignancy are distinct but interactive entities.

Surgery can be performed before and/or after delivery [5]. When surgery can be allowed, the treatment of patients diagnosed in the late third trimester might be delayed until postpartum.

In patients with recent vulvar scarring or following plastic reconstruction surgery, or in case of large VSCCs at increased risk of bleeding during vaginal birth, CS should be favored [5]. An elective CS can be performed to prevent vulvar wound dehiscence after vulvar surgery for VSCC, but vaginal delivery cannot be excluded as an option especially in case of small, well-healed vulvar wounds or maybe in case of a small VSCC [5]. Dissemination of tumor cells caused by mechanical dilation of vulva during labor is hypothetically possible, but there is no clear evidence that vaginal delivery may increase the risk of VSCC recurrence: as only 37 patients were reported, larger series are required.

Despite possible risks on pregnancy outcomes and fetal mortality/morbidity, some patients of our series underwent invasive treatment during pregnancy, including extensive lymphadenectomy, SLN resection, radiotherapy, or chemotherapy. In the reported cases, all these types of treatment seemed not to significantly affect the pregnancy/fetal outcomes, as the mothers of the only reported stillborn [36] and resuscitated baby [22] were not treated during pregnancy. The reported treatment complications were usually local (lymphoceles, wound breakdown, abscess/mycosis, hematomas), fortunately without significant impact on the babies. However, the possibility of the development of a systemic infection can not be completely avoided. As to the increased gestational vulvar blood flow (especially in the third trimester), surgery may result in higher blood loss, which can delay the postpartum treatment of cases diagnosed after 36 GW: judicious electrocautery can reduce the blood loss [5].

As per the NCCN-g, SLN biopsy results in decreased postoperative morbidity than IF-LND, which is associated with wound complication (20–40%) or lymphedema (30–70%) [43]: as per our review, the 4 patients who undergo SLN procedure did not have complications, but few cases were tested to allow significant considerations. Fetal exposure to locally injected ^99m^Tc (0.25 mCi, T1/2: 6 h) can be reduced by short treatment protocols, performing this procedure 2 h after injection using the lowest possible dose [5]. Systemic exposure to ^99m^Tc seems insignificant, as the isotope is captured in the SLN which is going to be removed. Both lymphoscintigraphy/SPECT (Single Photon Emission Computed Tomography) and Blue dye (for possible anaphylaxis) should be omitted during pregnancy [5]. SLN resection was performed during pregnancy in 2 cases [12,17]: in 1 case, ^99m^Tc (dosages of 10.73 and 10.15 and 11.07 and 9.9 MBq on 4 sites of injection) was used to identify SLN by scintigraphy [17]. In another case, isosulfan blue was used postpartum to identify bilateral SLN declining ^99m^Tc for patient’s concern about possible radioactive exposure while breast-feeding [18].

During pregnancy, radiotherapy and chemotherapy are contraindicated. Very few patients of our series were treated, apparently without significant effects on pregnancy/fetal outcomes. During this period, if feasible, it’s better to achieve the surgical therapy best fitted with the patients’ characteristics. When indicated, adjuvant radiotherapy should start soon: to allow delivery, delay of radiotherapy by 6–8 weeks is within safety limits [5,6]. Anyway, data on the efficacy/side effects of adjuvant radiotherapy are scant as to the rarity of VSCCs diagnosed during pregnancy; there are no clearly reported guidelines [6]. Neoadjuvant chemotherapy to reduce tumor size for locally advanced disease remains experimental [5,6].

VSCCs may show different histological patterns of invasion ranging from infiltrating islands/nests to more complex, exophytic/expansile growth; verrucous, warty (condylomatous), basaloid, acantholytic (adenoid or pseudoglandular), or spindle cell (pseudosarcomatous) features with prominent fibromyxoid stroma were also described [1]. Mixed patterns can be found [1]. Keratinization may be variable, depending on grade and subtype [1]. Verrucous carcinomas are part of the spectrum of HPV-independent carcinomas [1]: they are well differentiated (variable keratinization, minimal nuclear atypia, abundant eosinophilic cytoplasm, non-atypical mitoses), showing cohesive/verruciform growth with bulbous pegs and broad invasive front.

Currently, there is no difference in treatment between HPV-associated (1/3 cases) and HPV-independent (2/3 cases) VSCCs, but the WHO recommends classifying them in the pathology report, as HPV-independent VSCCs behave more aggressively [1]. The histomorphological features of 2 VSCC-subtypes significantly overlap and cannot be confidentially distinguished on conventional hematoxylin and eosin histological slides [1,45]. Ideally, validated molecular tests should confirm the presence of HPV [1]. HPV16 is involved in > 70% of all HPV-associated VSCCs while low-risk HPV-genotypes (6, 11, etc.) are exceptionally identified [1]. Only a case of our series was tested for HPV: high-risk HPV-subtypes (16, 18, 31, 33, 35, 45, 51, 52, 56) were identified [27].

The search for easily available and cost-effective surrogate markers for molecular analysis/HPV tests have shifted to alternative methods such as immunohistochemistry [1,45,46]. Immunohistochemical block-type p16-positivity (strong, diffuse and continuous in basal layers with variable extension to the superficial layers) is a reliable (although imperfect) surrogate marker of HPV-associated VSCCs: viral oncoproteins E6-E7 cause p53/RB1 degradation leading to p16-overexpression [1,45]. Conversely, p16 is usually negative or non–block in HPV-independent VSCCs [1].

p53 is usually expressed with a wild-type pattern in HPV-related VSCCs, despite TP53 somatic mutations can sometimes occur [1,45,47]. Conversely, more than two-thirds of HPV-independent VSCCs show immunohistochemical p53 overexpression (missense mutation) or lack expression (truncating stop/gain mutations) [1,45]. p53 immunohistochemical patterns showed excellent correlation with TP53 mutational status [45,47]. Four mutation-type patterns can be reproducibly found: basal overexpression; basal and parabasal/diffuse overexpression (most common); completely absent staining (“null” staining); cytoplasmic staining (with variable nuclear expression) [45,47]. Conversely, 2 wild-type p53 immunohistochemical patterns were identified (“scattered” expression; basal-sparing mid-epithelial staining) [45,47]. In the recent series of Tessier-Cloutier et al., these patterns were consistent with TP53 mutation status in 58/61 (95%) VSCCs and 39/42 (93%) vulvar in situ lesions [47].

The p16/p53 expression profile seems to have prognostic implications, helping the identification of HPV-related VSCCs, which show a more favorable outcome. Moreover, recent findings suggested that HPV-negative/p53 wild-type VSCCs may have an intermediate prognosis between HPV-positive/p53 wild-type VSCCs and HPV-negative/p53-mutated VSCCs (worse outcome): larger studies are needed [48]. Unfortunately, immunohistochemical examination (also including p53 and p16 immunomarkers) was not performed in any of the VSCCs of our series.

NOTCH1/2, HRAS, or PIK3CA activating mutations were frequently reported in VSCCs [1], but none of the cases included in our review was tested.

Evidence of precursor lesions (HPV-associated SIL; HPV-independent VIN/differentiated VIN; inflammatory conditions) may help the correct diagnosis [1]. While HPV-independent tumors comprise the majority of VSCCs, HPV-independent VIN forms only a small minority (2–3.5%) of all solitary VIN/SIL diagnoses. HPV-negative precursors may be clinically more subtle (less likely to be symptomatic and biopsied) or more difficult to be recognized on histological examination by pathologists; another option is that they may progress quickly to VSCCs (which can replace the precursor areas) [49].

However, morphologic evaluation shows limitations in predicting the HPV status of precursor lesions: histologically ambiguous lesions were described [1,45,49,50,51,52]. Some HPV-negative VINs showed a basaloid histologic pattern similar to that of HPV-associated H-SILs [50]. Conversely, Rakislova et al. identified differentiated VIN-like and lichen sclerosus-like lesions associated with HPV-related VSCCs [51]. Moreover, Watkins et al. found that HPV-associated H-SIL with superimposed lichen simplex chronicus may mimic HPV-independent VIN (differentiated VIN) with possible abnormal basal p53 expression [52]. In our series, the evaluation of the precursor lesions was morphological, with potential diagnostic pitfalls: immunohistochemistry was not performed in any cases.

Benign conditions/tumors (decidualized endometriosis, seborrhoic keratosis, VCs, mammary-type glandular lesions, Bartholin gland lesions/cysts, etc.) [1,53,54], vulvar direct invasion or metastasis from SCCs arising elsewhere (cervix, vagina, anus, urinary tract, lungs, etc.) [1,55], or other primary or secondary malignancies may be considered in the differential diagnosis by clinicians. Other primary vulvar carcinomas diagnosed during pregnancy included choriocarcinoma (1 case) [56], salivary gland-type carcinomas (3 cases) [57,58,59], and adenocarcinoma of Bartholin’s gland (1 case) [60]. Salivary gland-type carcinomas included a myoepithelial carcinoma [57] and 2 adenoid cystic carcinomas [58,59]; also, according to our previous reviews, no other salivary gland-histotypes seemed to arise in the vulva during pregnancy [61,62,63]. Our review did not find vulvar carcinosarcomas arising in pregnancy.

For pathologists, the diagnosis is usually straightforward in typical cases despite it’s sometimes impossible to establish tumor origin in small biopsies not showing in situ areas. In carcinomas with squamous differentiation (endometrial, urothelial, etc.), a typical non-squamous component is usually present. Verrucous or warty (condylomatous) carcinomas may be misinterpreted as benign or non-invasive carcinomas. In fact, stromal invasion may be challenging to identify in superficially invasive tumors or biopsy material [1]. Desmoplasia, irregularly shaped nests (sometimes disconnected from the surface), loss of polarity and cytoplasmic eosinophilia of invasive cells favor invasion [1]. Four vulvar lesions were evident in pre-conceptional period (1–15 months before presentation): one was diagnosed as a condyloma 6 months before conception, while subsequent biopsy at 29 GW revealed a VSCC [25]. Another patient received a diagnosis of a L-SIL/H-SIL (“VIN1-2”) during pregnancy: as she and her family were non-compliant, it took time to obtain a new biopsy, revealing VSCC [26].

Most VSCCs are asymptomatic, sometimes unrecognizable by obese or pregnant patients with reduced mobility and dilated abdomen: 5/37 (13%) cases were identified during delivery. Some women experienced pruritus, burning sensation, pain, or bleeding, especially in association with vulvar dermatoses.

Patients and clinicians must not underestimate the emergence of new vulvar lesions during pregnancy, especially in women with risk factors (HPV infection, vulvar dermatosis, etc.). New lesions, even if small, should be biopsied and patients must be followed-up.

Stage and LN-status (number of involved nodes, size of metastasis, and extranodal growth) are the most important prognostic factors: a rapid diagnosis may allow the more appropriate treatment [1]. Depth of invasion is associated with LN-involvement and disease recurrence [1]. To SEER, the 5-year relative survival rate (5-yr SR) is 70.4% [3]. Prognosis of early-stage/localized VSCCs (60% of cases) is quite good (5-yr SR 80–90%) [1,3], decreasing with higher stage: 5-yr SR of “regional” (28%) and “distant” (6%) disease spread at presentation are 50.6% and 20.3%, respectively [1,3]. In our series, 57% of cases were stage I [12,14,17,18,22,23,24,25,28,29,30,33,34,36,37,38,39,40], 5% stage II [35,41], 30% stage III [11,15,16,19,21,26,27,28,31,35], 8% stage IV [13,20,32]. Eight women died of disease (22%) within 4 years: all the patients presented with high-stage disease (3 stage IV [13,20,32], 4 stage III [15,19,26,31]) except for a stage IB VSCC which was untreated and progressed [25].

Unfortunately, VSCC recurrence rates are globally high (12–37%) (32% in our series) [1]. HPV-associated VSCCs have better progression-free survival; other predictive recurrence factors include non-radical resection or tumor-free margins <3 mm, tumor size, lymphovascular or perineural invasion: it was not possible to clearly stratify VSCCs of our series according to these frequently unreported parameters.

## 4. Materials and Methods

A systematic literature review was performed according to the PRISMA guidelines [64], searching in multiple databases as previously described [61,62,65]. The study aimed to answer the following PICOS (Population, Intervention, Comparison, Outcomes) questions

1. Population: patients with a diagnosis of VSCC during pregnancy;
2. Intervention: any type of treatment, including surgery, chemotherapy, radiotherapy, or observational treatment;
3. Comparison: no comparisons are expected;
4. Outcomes: (1) patient’s status at last follow-up: no evidence of disease, alive with disease, dead of disease; (2) pregnancy outcome: healthy baby; stillborn.

Study design: retrospective observational study (case series, case reports).

Eligibility/inclusion criteria: studies describing patients with a diagnosis of VSCC during pregnancy; review articles were excluded.

Exclusion criteria: other carcinomatous histotypes; non-carcinomatous tumors (sarcomas, lymphomas, melanomas, etc.); tumors not arising from the vulva; cases with uncertain diagnosis.

Information sources and search strategy: we searched for (pregnancy OR pregnancy-associated OR pregnant OR gravid OR abortus OR abortion) AND (vulva OR vulvar) AND (carcinoma OR carcinomas OR adenocarcinoma OR adenocarcinomas OR cancer OR carcinosarcoma OR carcinosarcomas OR “malignant mixed mullerian”) in Pubmed (all fields) (https://pubmed.ncbi.nlm.nih.gov/ (accessed on 5 December 2020)), Web of Science (Topic/Title) (https://login.webofknowledge.com (accessed on 5 December 2020)) and Scopus (Title/Abstract/Keywords) (https://www.scopus.com/home.uri (accessed on 5 December 2020)) databases. No limitations or additional filters were set. Relevant articles were obtained in full-text format and screened for additional references. The bibliographic research ended on 30 January 2021.

Study selection: 2 independent reviewers (Andrea Palicelli, Vincenzo Dario Mandato) selected the studies using a 2-steps screening method. In the first step, the screening of titles and abstracts was performed to verify eligibility/inclusion criteria and exclude irrelevant studies. In the 2nd step, full texts of relevant articles were screened by the 2 reviewers to: (1) verify study eligibility and inclusion criteria and (2) avoid duplications of the included cases. Two other authors (Magda Zanelli, Laura Ardighieri) performed a manual search of reference lists to search for additional relevant publications. Loredana De Marco checked the data extracted.

The objective of the systematic review was as follows: (1) to update and summarize the literature concerning VSCCs diagnosed in pregnancy; (2) to report any information regarding clinic-pathological features, treatment strategies, and patients’ outcomes.

Data collection process/data items: data collection was study-related (authors and year of study publication) and case-related (patient age, clinical history/presentation, tumor gross and histological features, tumor stage at presentation, treatment, follow-up, and outcomes).

Statistical analysis: the collected data were reported as continuous or categorical variables. Categorical variables were summarized by frequency and percentage; continuous variables were summarized by ranges and mean and median values where appropriate. Time-to-recurrence was the time from primary treatment to disease recurrence. The survival status was the time from primary treatment to the last follow-up.

## 5. Conclusions

In conclusion, VSCCs are exceedingly rare during pregnancy. Patients and clinicians must not underestimate the arising of new vulvar lesions, especially in pregnant women with risk factors (HPV infection, vulvar dermatosis, etc.): even if they are small, they should be biopsied, and the patients must be followed-up.

When surgery is allowed, treatment of patients diagnosed in the late 3rd trimester might be delayed until postpartum. An elective CS can be performed to prevent vulvar wound dehiscence, but vaginal delivery is an option especially in case of small, well-healed vulvar wounds after vulvar surgery for VSCC. Despite possible risks on pregnancy outcomes and fetal mortality-morbidity, some patients of our series underwent invasive treatment during pregnancy: in the few reported cases, the pregnancy/fetal outcomes seemed not affected by invasive treatments. However, clinicians must be careful: larger cohorts should define the best therapy approach for this rare and unfortunate condition. In the absence of definite guidelines, multidisciplinary approach and discussion with patients are mandatory to tailor therapy according to the tumor, pregnancy, and patient characteristics.

The molecular features and HPV status of VSCCs may be relevant in the future to diagnose and treat VSCC in pregnant women.

## Figures and Tables

**Table 1:** Primary vulvar squamous cell carcinomas: macroscopic features.

Authors	Age	Site	Size (cm)	Gross Examination
Gitsham et al., 2020 [11]	40	NR	NR	NR
Metke et al., 2019 [12]	39	NR	NR	NR
Lecointre et al., 2015 [13]	29	Periclitoral, Lmi, LMA (mainly right)	6.7	Ulcerated
Hasanzadeh et al., 2014 [14]	37	Lmi, left	3	Ulcerated
Idi et al., 2013 [15]	32	LMA, right (site of previous excision)	15	Nodular, bleeding
Pariyar et al., 2012 [16]	20	NR	NR	NR
Nijman et al., 2012 [17]	27	Lmi, right	1.2	Painful, verrucous, ulcerated
Parva et al., 2009 [18]	29	Posterior fourchette	1	Raised, pigmented
Keskin et al., 2008: case 3 [19]	31	LMA, right, extending to the clitoris	3	NR
Ghosh et al., 2004 [20]	41	(1) Lower part of right labia, eroding posterior fourchette, lower vagina and anal canal (2) LMA, right	(1) ~4 (2) 2	(1) Solid, necrotic (2) Exophytic ulcerated
Modares Gilani et al., 2005 [21]	28	Clitoris, upper Lmi and LMA	8	Ulcerated, verrucous, necrotic
Alexander-Sefre et al., 1998 [22]	18	LMA, right (3 mm from the clitoris)	1	Ulcerated, white, firm, freely mobile plaque
Ogunleye et al., 2004 [23]	36	(1) Anterior to the left LMA (2) Posterior to the right of the clitoris (*)	(1) 3.5 (2) 0.3	(1) Painful, tender (2) Tender, raised
Couvreux-Dif et al., 2003: case 1 [24]	34	Lmi, left anterior	1	Painful, raised
Couvreux-Dif et al., 2003: case 2 [24]	31	(1) Lmi, right (11 o’clock) (2) Lmi, left (*)	(1) 2 (2) NR	(1) Nodule; (2) NR
Olayemi et al., 2002 [25]	29	Whole vulva (more on the left part)	6	Cauliflower-like, exophytic
Bakour et al., 2002 [26]	29	Clitoris	2	Indurated ulcerated, then more edematous and necrotic.
Heller et al., 2000 [27]	28	Lmi, left	4	Painful, ulcerated
Gitsch et al., 1995: case 1 [28]	29	Bilateral Lmi, anterior and periclitoral (extension within 1 mm of the urethra)	5	Ulcerated
Gitsch et al., 1995: case 2 [28]	35	Multifocal: most vulva (especially posterior 2/3), fourchette	NR	Red-white papillary
Regan et al., 1993 [29]	37	LMA, left upper half	2	Ulcerated
Del Priore et al., 1992 [30]	39	Periclitoral	NR	Ulcerated
Moore et al., 1991: case 1 [31]	24	(1) Clitoris and LMA, upper left (2) LMA, lower right	(1) 5 (2) 1	Abscess-like, raised
Moore et al., 1991: case 2 [31]	35	LMA, right lower third	1	NR
Sivanesaratnam et al., 1990 [32]	28	Periclitoral (then upper half of bilateral LMA, clitoris, lower mons pubis)	2	Fan-shaped, exophytic
Robson et al., 1989 [33]	33	LMA, left	NR	Ulcerated
Rahman et al., 1982 [34]	28	LMA, left upper third, involving the clitoris	3	Proliferative
Kempers et al., 1965: case 1 [35]	25	Posterior vulva	large	Fungating, ulcerated
Kempers et al., 1965: case 2 [35]	32	Posterior vulva and perineum	large	Fungating
Collins et al., 1963: case 3 [36]	28	Lmi, left (upper third)	2	Ulcerated
Collins et al., 1963: case 4 [36]	26	(1) Lmi, right (upper 2/3) (2) LMA, left	(1) 5 (2) small	(1) Exophytic (2) Ulcerated
Collins et al., 1963: case 5 [36]	31	LMA, right (middle third)	4	Flat, hard
Barber et al., 1963: case 3 [37]	27	LMA, bilateral	NR	NR
Gemmell et al., 1962: case 10 [38]	30	Clitoris	small	Ulcerated
De Bruine TLA, 1958 [39]	32	Clitoris, extending in all directions	4	Ulcerated
Shannon et al., 1941 [40]	26	LMA, right	6	Fungating, ulcerated
Russell et al., 1940 [41]	17	LMA, left	4	Raised

(*): subsequent/metachronous. Lmi: Labium minus; LMA: Labium majus; NR: not reported.

**Table 2:** Pathological features of vulvar carcinomas and precursors.

Authors	Grade	Stage (°)	HPV-Related Lesions	Inflammatory Conditions
Gitsham et al. [11]	G2	IIIA	NR	NR
Metke et al. [12]	NR	IB	NR	NR
Lecointre et al. [13]	G3	IVB	NR	Recurrent vulvar psoriasis
Hasanzadeh et al. [14]	NR	IB	NR	VH + CD (history)
Idi et al. [15]	G3	IIIA	NR	NR
Pariyar et al. [16]	NR	III	NR	NR
Nijman et al. [17]	G1	IB	no HPV	Lichen sclerosus
Parva et al. [18]	G1	IB	H-SIL (VIN2)	Lichen sclerosus
Keskin et al.: case 3 [19]	G1	IIIA	NR	NR
Ghosh et al. [20]	G3	IVB(m)	NR	NR
Modares Gilani et al. [21]	G1	IIIA	VCs/HPV (history)	NR
Alexander-Sefre et al. [22]	G1	IA	No SIL	HLP + HVD
Ogunleye et al. [23]	G2	IB(m)	H-SIL (VIN3)	Lichen sclerosus
Couvreux-Dif et al.: case 1 [24]	G1	IA	NR	Lichen sclerosus
Couvreux-Dif et al.: case 2 [24]	G1	IB(m)	VCs, H-SIL (bowenoid VIN)	NR
Olayemi et al. [25]	NR	IB (@)	VC (?)	NR
Bakour et al. [26]	G3	IIIA	NR	NR
Heller et al. [27]	G2	IIIA	VCs, H-SIL (cervix, vulva, anus)	NR
Gitsch et al.: case 1 [28]	G2	IB	NR	NR
Gitsch et al.: case 2 [28]	NR	IIIA(m)	VCs/HPV, H-SIL (VIN3, cervix), VW	NR
Regan et al. [29]	G2	IB	VCs, PBD (#)	NR (#)
Del Priore et al. [30]	G2	IB	NR	Lichen sclerosus
Moore et al.: case 1 [31]	G2	IIIB (m)	H-SIL (VIN3)	NR
Moore et al.: case 2 [31]	G2	IIIB	NR	NR
Sivanesaratnam et al. [32]	G1 (§)	IVB	NR	NR
Robson et al. [33]	G2	I	NR	NR
Rahman et al. [34]	G1	IB	NR (#)	NR (#)
Kempers et al.: case 1 [35]	G3	IIIB	NR	NR
Kempers et al.: case 2 [35]	G1	II	NR	NR
Collins et al.: case 3 [36]	NR	IA	NR	NR
Collins et al.: case 4 [36]	NR	IB(m)	VCs, vulvar H-SIL (CIS), PCs	NR
Collins et al.: case 5 [36]	NR	IB	NR	NR
Barber et al.: case 3 [37]	NR	I	NR	NR
Gemmell et al.: case 10 [38]	G1	IA	NR (#)	NR (#)
De Bruine TLA [39]	NR	IB	NR	Severe kraurosis vulvae
Shannon et al. [40]	NR	IB	VCs	NR
Russell et al. [41]	NR	II	NR	NR

Table S3 shows further details. (°): estimated stage (AJCC classification, 8th ed.) [2] (the real stage is at least the estimated stage); (§): G3 spindle cells in metastatic lymph nodes; (@): without treatment, it progressed to stage IVB; (?): The lesion which turned to be a carcinoma was diagnosed as vulvar condyloma 6 months before conception; (#): vulvar leukoplakia was present but the underlying histological lesion was unclear. CIS: carcinoma in situ; HLP + HVD: hypertrophic lichen planus and hyperplastic vulvar dystrophy without atypia; HPV: human papillomavirus; H-SIL: high-grade squamous intraepithelial lesion; m: multifocal; NR: not reported; PBD: Bowen disease of perineal skin; PCs: perianal condylomas; SIL: squamous intraepithelial lesion; VC: vulvar condyloma; VH + CD: vulvar hyperplasia + chronic dermatitis; VIN; vulvar intraepithelial neoplasia; VW: vaginal warts.

**Table 3:** Follow-up data.

Authors	Recurrence	Follow-Up
Gitsham et al. [11]	Local (6 mo) (wide excision + left IF-LND + local RT)	AWD (6 mo)
Metke et al. [12]	No	NED (6 mo)
Lecointre et al. [13]	PD dp (vulva: 13 + 2 we ad; lungs: 5 mo ad)	DOD (cardiorespiratory decompensation for pulmonary MTS) (5 mo)
Hasanzadeh et al. [14]	No	NED (24 mo)
Idi et al. [15]	PD (right inguinal) (no treatment; lack of RT)	DOD (5 mo)
Pariyar et al. [16]	NR	NR
Nijman et al. [17]	No	NED (12 mo)
Parva et al. [18]	(1) Subcutis (6 mo) (excision with negative margins); (2) Subcutis (NR) (FNA + ChT/RT)	AWD (>6 mo)
Keskin et al.: case 3 [19]	Inguinal lymph nodes (36 mo) (salvage therapy)	DOD (48 mo)
Ghosh et al. [20]	PD (bilateral lower limb edema, enlarged groin nodes) (3 mo) (declined ChT)	DOD (11 mo)
Modares Gilani et al. [21]	No	NED (7 mo)
Alexander-Sefre et al. [22]	No	NED (4 mo)
Ogunleye et al. [23]	No (*)	NED (16 mo)
Couvreux-Dif et al.: case 1 [24]	No	NED (33 mo)
Couvreux-Dif et al.: case 2 [24]	No (*)	NED (19 mo)
Olayemi et al. [25]	PD (abdomen, anus, inguinal) (15 we ad) (*) (i.v. fluids, antibiotics, blood transfusions, suprapubic cystostomy)	DOD (sepsis) (4 mo)
Bakour et al. [26]	PD (new inguinal lesions) (after RT completion) (palliative)	DOD (9 mo)
Heller et al. [27]	NR	NED or AWD (lost at FU)
Gitsch et al.: case 1 [28]	No	NED (39 mo)
Gitsch et al.: case 2 [28]	Periclitoral maculo-papular eruption (23 mo) (biopsy + anterior superficial vulvectomy) (VIN3; VIN2 on surgical margin)	NED (28 mo)
Regan et al. [29]	NR	NED or AWD (lost at FU)
Del Priore et al. [30]	NR	NR
Moore et al.: case 1 [31]	No	NED (16 mo)
Moore et al.: case 2 [31]	Lumbar vertebrae, periaortic lymph nodes (16 mo) (palliative RT)	DOD (27 mo)
Sivanesaratnam et al. [32]	PD (NR)	DOD (sepsis) (2.5 mo)
Robson et al. [33]	No	NED (6 mo)
Rahman et al. [34]	No	NED (24 mo)
Kempers et al.: case 1 [35]	No	NED (96 mo)
Kempers et al.: case 2 [35]	No	NED (48 mo)
Collins et al.: case 3 [36]	No	NED (54 mo)
Collins et al.: case 4 [36]	Bilateral sides of introitus (12 mo) (wide excision of 5 vulvar and perineal areas; diagnosis: superficially-invading SCC, CIS, MAEH) Cervical stage 2 SCC (9 years; RT). Small vaginal areas of CIS (16 years) (wide excision)	NED (204 mo)
Collins et al.: case 5 [36]	No	NED (92 mo)
Barber et al.: case 3 [37]	No	NED (72 mo)
Gemmell et al.: case 10 [38]	No	NED (53 mo)
De Bruine TLA [39]	No	NED (50 mo)
Shannon et al. [40]	No	NED (17 mo)
Russell et al. [41]	No	NED (60 mo)

(*): but new metachronous primaries were identified, both diagnosed during pregnancy: the interval of time from excision of the 1st lesion and the subsequent tumor was 7 [24] and 11 weeks [23]. ad: after diagnosis; AWD: alive with disease; ChT: chemotherapy; CIS: carcinoma in situ; DOD: dead of disease; dp: during pregnancy; FNA: fine-needle aspiration; FU: follow-up; mo: months; i.v.: intravenous; IF-LND: inguinofemoral lymphadenectomy; MAEH: leukoplakia with markedly atypical epithelial hyperplasia; MTS: metastases; NED: no evidence of disease; NR: not reported; PD: progression of disease; RT: radiotherapy; SCC: squamous cell carcinoma; VIN: vulvar intraepithelial neoplasia; we: weeks.

**Table 4:** Details of pregnancies.

Authors.	Age	Pregnancy History	Time of Delivery	Delivery-Type	Relevant Events of Ongoing Pregnancy	Baby Weight (gr)
Gitsham et al. [11]	40	G3P2A1	37 GW + 2 d	CS	NR	NR
Metke et al. [12]	39	NR	NR	NR	NR	NR
Lecointre et al. [13]	29	G2P0A2 (#)	29 GW + 1 d	CS (emergency for sepsis)	Sepsis (*Pseudomonas aeruginosa*) of vulvar origin, uncontrolled by antibiotics	1296
Hasanzadeh et al. [14]	37	G8P6 (§)	term	CS	NR	NR
Idi et al. [15]	32	G6P5 (1 EPr 3 years before)	NR	CS	NR	NR
Pariyar et al. [16]	20	G1P0	NR	VA	NR	NR
Nijman et al. [17]	27	NR	38 GW + 3 d	CS	Obstetrical reasons lead to CS	NR
Parva et al. [18]	29	G0P0	32 GW	CS (elective, after 1 course of steroids for lung maturation)	no	1928
Keskin et al.: case 3 [19]	31	G3P2	NR (>31)	CS	no	NR
Ghosh et al. [20]	41	G2P1(*) A1	term + 7 d	CS (lower segment)	Prolonged pregnancy	3320
Modares Gilani et al. [21]	28	G6P5	36 GW	CS	no	2800
Alexander-Sefre et al. [22]	18	G0P0	29 GW	VA	(@)	1000
Ogunleye et al. [23]	36	G5P4	37 GW	VA (induced)	Subsequent focus of VSCC (34 GW)	NR
Couvreux-Dif et al.: case 1 [24]	34	G4P3	38 GW	VA + right lateral EP	no	3750
Couvreux-Dif et al.: case 2 [24]	31	G4P3	38 GW	CS	Subsequent focus of VSCC (22 GW, 2 weeks after surgery); VC (right labium minus, 27 GW); vulvar scars; abnormal fetal heartbeat; membrane rupture	2300
Olayemi et al. [25]	29	G2P2 (none alive)	37 GW	CS (elective)	no	2250
Bakour et al. [26]	29	G1P1 (°)	38 GW	CS (elective)	Uterine fibroids Small antepartum hemorrhage	NR
Heller et al. [27]	28	G5P5	NR	CS	NR	NR
Gitsch et al.: case 1 [28]	29	G4P3	40 GW	CS	CS for vulvar scars	NR
Gitsch et al.: case 2 [28]	35	G4P3	35 GW	CS	NR	NR
Regan et al. [29]	37	G3P1A2	38 GW	VA + midline EP (no laceration or extension)	*Chlamydia* infection (treated) Cocaine and heroin abuse	2780
Del Priore et al. [30]	39	G8P4A4	38 GW	VA (#) + midline EP extended into 4th degree laceration	Suppurative vulvar cellulitis (intravenous antibiotics) (25–27 GW)	3990
Moore et al.: case 1 [31]	24	G0P0	36 GW	CS + I/E-I-LNS + right oophoropexy to right paracolic gutter	Amniocentesis: lecithin–sphingomyelin ratio >2.0; presence of phosphatidyl glycerol.	2670
Moore et al.: case 2 [31]	35	G9P6A3	term	VA (#)	NR	NR
Sivanesaratnam et al. [32]	28	G2P1	term	VA (#)	NR	3500
Robson et al. [33]	33	G0P0	>24 GW	CS	no	NR
Rahman et al. [34]	28	G7P6	term	VA + right mediolateral EP	no	3400
Kempers et al.: case 1 [35]	25	G4P4	NR	VA	NR	NR
Kempers et al.: case 2 [35]	32	G7P6	37 GW	VA (#) + bilateral EPs	no	NR
Collins et al.: case 3 [36]	28	G8P6A2	37 GW	low-forceps under spinal anesthesia + PC	no	2268
Collins et al.: case 4 [36]	26	G4P2	8 mo	VA (#)	NR	NR
Collins et al.: case 5 [36]	31	G7P6	NR	VA (#) + left mediolateral EP	Stillborn	NR
Barber et al.: case 3 [37]	27	G2P2	NR	CS + completion surgery	NR	NR
Gemmell et al.: case 10 [38]	30	G0P0	9 mo	CS (hemorrhage)	Hypertension and proteinuria at 34 GW; hemorrhage at 36–37 GW	2295
De Bruine TLA [39]	32	G0P0	9 mo	CS (lower segment)	Progesterone therapy to protect pregnancy during surgery	3490
Shannon et al. [40]	26	G2P0A2	8 mo	CS	no	2807
Russell et al. [41]	17	G0P0	8 mo	CS	(ç)	NR

(#): spontaneous. (§): 2 perinatal deaths. (*): the newborn died 3 months after delivery for sudden infant death syndrome. (°): during previous pregnancy, the patient experienced moderately/severe, transient bone marrow hypoplasia confirmed by bone marrow aspiration at 36 gestational week; then, 10 years of secondary subfertility. (@): At delivery, the baby was resuscitated and transferred to the special baby care unit. (ç): Postpartum pyelitis, abscess of left axillary space for 15 days. A: abortus; CS: cesarean section; d: days; EP: episiotomy; EPr: ectopic pregnancy; G: gravida; GW: gestational week; I/E-I-LNS: sampling of internal and external iliac lymph nodes; mo: months; NR: not reported; P: parity; PC: posterior colporrhaphy; VA: vaginal; VC: vulvar condyloma; VSCC: vulvar squamous cell carcinoma.

## Supplementary Materials

The following are available online at https://www.mdpi.com/2072-6694/13/4/836/s1, Table S1. Presentation of vulvar squamous cell carcinomas during pregnancy, Table S2. Treatment of vulvar carcinomas in pregnancy: further details, Table S3. Pathological features of vulvar carcinomas and precursors: further details.
