# Supplementary material for: How Can We Treat Vulvar Carcinoma in Pregnancy? A Systematic Review of the Literature
Source: Cancers (Basel). 2021 Feb 17;13(4):836. doi: 10.3390/cancers13040836 (PMC7921964; doi:10.3390/cancers13040836)
Supplement: Supplementary file 1 [file cancers-13-00836-s001.pdf]

cancers

MDPI

Supplementary Materials: How Can We Treat Vulvar Carcinoma in Pregnancy? A Systematic Review of the Literature

Andrea Palicelli, Lucia Giaccherini, Magda Zanelli, Maria Paola Bonasoni, Maria Carolina Gelli, Alessandra Bisagni, Eleonora Zanetti, Loredana De Marco, Federica Torricelli, Gloria Manzotti, Mila Gugnoni, Giovanni D'Ippolito, Angela Immacolata Falbo, Filomena Giulia Sileo, Lorenzo Aguzzoli, Valentina Mastrofilippo, Martina Bonacini, Federica De Giorgi, Stefano Ricci, Giuditta Bernardelli, Laura Ardighieri, Maurizio Zizzo, Antonio De Leo, Giacomo Santandrea, Dario de Biase, Moira Ragazzi, Giulia Dalla Dea, Claudia Veggiani, Laura Carpenito, Francesca Sanguedolce, Aleksandra Asaturova, Renzo Boldorini, Maria Giulia Disanto, Margherita Goia, Richard Wing-Cheuk Wong, Naveena Singh and Vincenzo Dario Mandato

1. Details of Some Additional Cases Excluded from Our Review

In 1977, Lutz et al. [7] found 5 vulvar carcinomas associated with pregnancy (3 VSCCs, 1 adenocarcinoma of the Bartholin's gland, 1 in situ carcinoma) (5.4% of vulvar malignancies during pregnancy; 1:8000 pregnant women): 3 were diagnosed and treated during the 2nd trimester of pregnancy, 2 within 6 months postpartum. The 5 patients were black, with an age range of 25–35 years. Only the patient with an in situ carcinoma remained alive. Two patients died 10 years (pulmonary metastases) and 18 years (mediastinal and pericardial metastases) after diagnosis. Another patient presented with metastases to the inguinal lymph nodes, distal urethra, and pubic periosteum: she died within 15 months after diagnosis. The last patient died from cardiac arrest while undergoing anterior exenteration. We excluded this article as some data were presented aggregated or lacking, being impossible to correlate prognosis and histotype.

In 1979, Shafeek et al. [8] reported a patient with vulvar warty lesions diagnosed as condylomas during pregnancy; however, a postpartum biopsy of the increased lesions involving the clitoris and the anterior half of labia majora revealed a grade 1, invasive VSSC: misdiagnosis of the 1st biopsy or malignant transformation (after delivery or during pregnancy) may explain this discrepancy. After radical vulvectomy and inguinal lymphadenectomy (pN+), she was lost at follow-up. As the diagnosis of VSCC was made postpartum, this case was excluded.

In 1982, Kitayama et al. [9] reported a VSCC in a 30-year-old woman: this article was written in Japanese language was irretrievable and thus excluded.

In 1993, Carter et al. [10] reported that 2/37 (5%) VSCCs of their series were associated with pregnancy in patients under age 40 years: further details were not provided.

Table S1. Presentation of vulvar squamous cell carcinomas during pregnancy.

**Involvement of Vulvar Sites (*)**	
Labium majus (49%)	Anterior/Upper vulva (60%)
Labium minus (30%)	Posterior/Lower vulva (27%)
Posterior fourchette (8%)	Whole vulva (5%)
Clitoris/periclitoral area (38%)	-
**Vulvar Lesions**	**Other Involved Sites**
Bilateral (49%)	Urethra (3%)
Unilateral (46%)	Vagina (8%)
Right-to-Left ratio: 0.9	Anus (8%)
Multifocality (16%)	Lower rectum (3%)
-	Palpable lymph nodes (33%)

(*) ≥1 vulvar sites may be involved by the same lesion.

Table S2. Treatment of vulvar carcinomas in pregnancy: further details.

Authors	Time of Diagnosis	PT-dp	PT-dp (Time)	Time of Delivery	PT-ad	PT-ad (Time)
Gitsham et al. [11]	36 GW	VB	-	37 GW +2 days	HV + bi-SLN + R-IF-LND	NR
Metke et al. [12]	32 GW	VB + S-NOS + bi-SLN	34 GW	NR	LE	NR
Lecointre et al. [13]	15 GW	VB + LN-FNAC + ChT (*)	18 GW	29 GW +1 day	ChT (**) + RT	9 days
Hasanzadeh et al. [14]	Discovery during 3rd month of pregnancy, diagnosis 2 weeks postpartum	No	-	term	VB + RV + bi-G-LND	NR
Idi et al. [15]	Diagnosis during 5th month of pregnancy in the site where a lesion was excised 1 month bc	No	-	NR	R-HV + R-I-LND	NR
Pariyar et al. [16]	20 GW	S-NOS (***)	Unclear	NR	S-NOS (***)	unclear
Nijman et al. [17]	Diagnosis during pregnancy (19 GW) but evident some months bc	VB + LE (°) + r-SLN	NR	38 GW +3 days	No	-
Parva et al. [18]	26 GW	VB + PV	28 GW	32 GW	RV + bi-SLN + R-G-LNS	3 weeks
Keskin et al.: case 3 [19]	31 GW	VB	-	NR (after 31)	RV + bi-IF-LND + RT	1 week
Ghosh et al. [20]	Term + 7 days (during CS)	No	-	term + 7 days	VB © + defunctioning colostomy (2 weeks postpartum) + palliative RT	4 weeks
Modares Gilani et al. [21]	33 GW	Topical antibiotics + EB	33 GW	36 GW	RV + bi-IF-LND + ChT/RT (§)	3 weeks
Alexander-Sefre et al. [22]	Discovery during delivery, diagnosis 29 weeks postpartum	No	-	29 GW	VB © + LE (°°)	3 weeks
Ogunleye et al. [23]	1) 18 GW; 2) 34 GW	VB + RV + bi-IF-LND	23 GW	37 GW	LE	9 weeks
Couvreux-Dif et al.: case 1 [24]	19 GW	VB + PV + L-I-LND	26 GW	38 GW	No	-
Couvreux-Dif et al.: case 2 [24]	1) 10 GW; 2) 22 GW	VB + PV + R-LND + L-HV (#) + biopsy of VC	15 GW + 22 GW	38 GW	No	-
Olayemi et al. [25]	During pregnancy (29 GW) but the lesion was evident 15 months bc and diagnosed as VC 6 months bc	VB	no (financial problems, denied treatment)	37 GW	no (financial problems, denied treatment)	no (financial problems, denied treatment)

Bakour et al. [26]	33 GW	VB n°1	-	38 GW	VB n°2 + RV + LD? + RT	6 weeks
Heller et al. [27]	Diagnosis during delivery (NR)	No	-	NR	VB© + RV + bi-I-LND + cone biopsy	12 weeks
Gitsch et al.: case 1 [28]	21 GW	VB + RV (@) + bi-IF-LND	22 GW	40 GW	No	-
Gitsch et al.: case 2 [28]	16 GW	VB + RV + bi-IF-LND	17 GW	35 GW	RT	6 weeks
Regan et al. [29]	24 GW	EB + RV + bi-I-LND	24 GW+ 28 GW	38 GW	No	
Del Priore et al. [30]	27 GW	VB + modified anterior RV	29 GW	38 GW	bi-SI-LND	6 weeks
Moore et al.: case 1 [31]	31 GW	VB	-	36 GW	I/E-I-LNS © + RV + VREC + bi-IF-LND	During delivery + after 2 weeks
Moore et al.: case 2 [31]	The lesion presented in the 2nd trimester (not biopsied till postpartum, NR)	No	-	term	VB + LN-FNAC (left groin) + RV + VREC + bi-IF-LND + RT	NR
Siva-nesaratnam et al. [32]	16 GW	no (lost at follow-up)	-	term	RV + bi-IP-LND	NR
Robson et al. [33]	18 GW	EB + RV + bi-G-LND	18 GW+ 21 GW	NR (after 24 GW)	NR	NR
Rahman et al. [34]	24 GW	VB + RV + bi-GF-LND	26 GW	term	No	-
Kempers et al.: case 1 [35]	Discovery during delivery (NR)	No	-	NR	VB © + RV + bi-FI-LND + RT	5 weeks
Kempers et al.: case 2 [35]	2nd month	VB + RV with excision of entire perineum + bi-IFEIO-LND	before 37 GW	37 GW	No	-
Collins et al.: case 3 [36]	6th month	VB + RV	28 GW	37 GW	bi-IFPAC-LND	15 days
Collins et al.: case 4 [36]	7th month	No	-	8 month	RV + bi-IFPAC-LND	11 days + 1 month
Collins et al.: case 5 [36]	Discovery during delivery (NR)	No	-	NR	VB © + RV + bi-IFPAC-LND	18 days + 29 days
Barber et al.: case 3 [37]	5th month	Vulvectomy	4 months before delivery	NR	Completion of vulvectomy + lymphadenectomy NOS	NR
Gemmell et al.: case 10 [38]	21 GW	EB + vulvectomy + resection of palpable left superficial lymph node	21 GW + 22 GW	9 month	No	-
De Bruine TLA [39]	4th month	VB + RV + bi-IF-LND (Rupprecht-Stoeckel type)	28 GW	9 month	No	-
Shannon et al. [40]	6.5th month	VB + simple vulvectomy	6.5 months	8 month	bi-IF-LND + RT	4 months

Russell et al. [41]	7th month	VB + RT (radium bombs)	after 7th month of pregnancy	8 month	RT + vulvectomy + inguinal lymphadenectomy	17 days +6 months

(*): weekly carboplatin-vinorelbine; (**): cetuximab + cisplatin; then only cetuximab; (***): unclear when performed; (°): sparing of the groins and clitoris; (°°): local excision including the clitoris; ©: during delivery; (§): 3 courses of Vincristin 1 mg/m^2 and Cisplatin 50 mg/m^2; (#): removing the second lesion and the clitoris at 22 gestational week); (@): with resection of 1 cm of the distal urethra. bc: before the probable conception; bi-FI-LND: bilateral femoral and iliac lymphadenectomy; bi-IFPAC-LND: bilateral superficial and deep inguinal femora, Cloquest's, external iliac, obturator, hypogastric, common iliac, aortic and caval lymphadenectomy; bi-G-LND: bilateral groin lymph node dissection; bi-GF-LND: bilateral groin and femoral lymphadenectomy; bi-I-LND: bilateral inguinal lymph node dissection; bi-IF-LND: bilateral inguinal-femoral lymph node dissection; bi-IFEIO-LND: bilateral inguinal, femoral, external iliac and obturator lymph node dissection; bi-IP-LND: bilateral inguinal and pelvic lymphadenectomy; bi-SI-LND: bilateral superficial inguinal lymphadenectomy; bi-SLN: bilateral sentinel lymph node; ChT: chemotherapy; CS: cesarean section; EB: Excisional biopsy; GW: gestational week; HV: hemivulvectomy; LD?: probable lymphadenectomy; I/E-I-LNS: sampling of internal and external iliac lymph nodes; LE: local excision; L-HV: left hemivulvectomy; L-I-LND: left inguinal lymph node dissection; LN-FNAC; fine needle aspiration of lymph node; NOS: not otherwise specified; PT-dp: Primary treatment during pregnancy; PT-ad: Primary treatment after delivery; PV: partial vulvectomy; R-HV: right hemivulvectomy; R-G-LNS: right groin lymph node sampling; R-I-LND: right inguinal lymphadenectomy; R-IF-LND: right inguinofemoral lymph node dissection; R-LND: right lymph node dissection; r-SLN: right sentinel lymph node dissection; RT: radiotherapy; RV: radical vulvectomy; S-NOS: surgery, not otherwise specified; VB: vulvar biopsy; VC: vulvar condyloma; VREC: vulvar reconstruction with tensor fascia lata muocutaneous flaps.

Table S3. Pathological features of vulvar carcinomas and precursors: further details.

Authors	TNM (°)	Additional Information
Gitsham et al. [11]	TxN1	-
Metke et al. [12]	T1bN0	-
Lecointre et al. [13]	T1bN1M1	The recurrent vulvar psoriasis was treated with topical corticoids at 5-12 gestational weeks
Hasanzadeh et al. [14]	T1bN0	Biopsy (2 years before): vulvar hyperplasia + chronic dermatitis
Idi et al. [15]	T1bN1	-
Pariyar et al. [16]	Stage 3	-
Nijman et al. [17]	T1bN0	Lichen sclerosus showed fusion of the labia, caudal of the clitoris.
Parva et al. [18]	T1bN0	Lichen sclerosus (history of 10 years) was treated with Clobetasol to control symptoms)
Keskin et al.: case 3 [19]	T1bN1	-
Ghosh et al. [20]	T3(m)cN + M1	-
Modares Gilani et al. [21]	T1bN1	Recurrent VCs/HPV infection in previous pregnancies (3-10 years before).
Alexander-Sefre et al. [22]	T1aNx	Leukoplakia (hypertrophic lichen planus and hyperplastic vulvar dystrophy without atypia)
Ogunleye et al. [23]	T1b(m)N0	Persistent, biopsy-proven lichen sclerosus (4 years): successful treatment with Clobetasol + follow-up
Couvreux-Dif et al.: case 1 [24]	T1aN0	Lichen sclerosus (right and left interlabial) was treated with corticosteroids
Couvreux-Dif et al.: case 2 [24]	T1b(m)N0	VCs (history, associated with primary lesion and multifocal metachronous), (labium minus, right)
Olayemi et al. [25]	T1bNx	-
Bakour et al. [26]	T2N1	-
Heller et al. [27]	T1bN1	VCs (entire vulva) at presentation

Gitsch et al.: case 1 [28]	T1bN0	-
Gitsch et al.: case 2 [28]	T1b(m)N1	VIN3, VCs (history of 16 years; at presentation most vulva was affected); HPV infection; H-SIL (cervix); vaginal warts.
Regan et al. [29]	T1bN0	VCs (history of 6 years); leukoplakia at presentation (thickened white vulvar epithelium and soft nodularities), Bowen disease of perineal skin.
Del Priore et al. [30]	T1bN0	Lichen sclerosus (10 years, biopsy-proven)
Moore et al.: case 1 [31]	T1b(m)N2	-
Moore et al.: case 2 [31]	T1aN2b	-
Sivanesaratnam et al. [32]	T2N2cM1	-
Robson et al. [33]	TxN0	-
Rahman et al. [34]	T1b	-
Kempers et al.: case 1 [35]	T2N2	-
Kempers et al.: case 2 [35]	T2N0	-
Collins et al.: case 3 [36]	T1aN0	-
Collins et al.: case 4 [36]	T1b(m)N0	VCs (left labium majus), vulvar CIS and perianal condylomas at presentation (upper anus)
Collins et al.: case 5 [36]	T1bN0	-
Barber et al.: case 3 [37]	TxN0	-
Gemmell et al.: case 10 [38]	T1aN0	-
De Bruine TLA [39]	T1bN0	Severe kraurosis vulvae (17 years) was treated with estrogens (topic and per os) + radiotherapy (9 doses; 300 rads/dose)
Shannon et al. [40]	T1bN0	Multiple bilateral VCs (labia majora) at presentation
Russell et al. [41]	T2N0	-

(°): estimated TNM stage according to the 8th edition of the AJCC classification [2] (the real stage is at least the estimated stage); CIS: carcinoma in situ; HPV: human papillomavirus; H-SIL: high-grade squamous intraepithelial lesion; VC: vulvar condyloma.
